# *NF1* mutations in conjunctival melanoma

**DOI:** 10.1038/s41416-018-0046-5

**Published:** 2018-03-21

**Authors:** S. L. Scholz, I. Cosgarea, D. Süßkind, R. Murali, I. Möller, H. Reis, S. Leonardelli, B. Schilling, T. Schimming, E. Hadaschik, C. Franklin, A. Paschen, A. Sucker, K. P. Steuhl, D. Schadendorf, H. Westekemper, K. G. Griewank

**Affiliations:** 1 Department of Ophthalmology, University Hospital Essen, Hufelandstrasse 55, Essen, 45147 Germany; 2 Department of Dermatology, University Hospital Essen, Hufelandstrasse 55, Essen, 45147 Germany; 3 Department of Ophthalmology, University Hospital Tübingen, Elfriede-Aulhorn-Strasse 7, Tübingen, 72076 Germany; 4 Department of Pathology, Memorial Sloan Kettering Cancer Center, 1275 York Avenue, New York, NY 10065 USA; 5 Institute of Pathology, University Hospital Essen, West German Cancer Center, University Duisburg-Essen and the German Cancer Consortium (DKTK), Hufelandstrasse 55, Essen, 45147 Germany; 6 Department of Dermatology, University Hospital Würzburg, Josef-Schneider-Straße 2, Würzburg, 97080 Germany; 7 Dermatopathologie bei Mainz, Bahnhofstr. 2b, Nieder-Olm, 55268 Germany

**Keywords:** Cancer genetics, Eye cancer

## Abstract

**Background:**

Conjunctival melanoma is a potentially deadly eye tumour. Despite effective local therapies, tumour recurrence and metastasis remain frequent. The genetics of conjunctival melanomas remain incompletely understood.

**Methods:**

A large cohort of 63 conjunctival melanomas was screened for gene mutations known to be important in other melanoma subtypes by targeted next-generation sequencing. Mutation status was correlated with patient prognosis.

**Results:**

Frequent mutations in genes activating the MAP kinase pathway were identified. *NF1* mutations were most frequent (*n* = 21, 33%). Recurrent activating mutations were also identified in *BRAF* (*n* = 16, 25%) and *RAS* genes (*n* = 12, 19%; 11 *NRAS* and 1 *KRAS*).

**Conclusions:**

Similar to cutaneous melanomas, conjunctival melanomas can be grouped genetically into four groups: *BRAF*-mutated, *RAS*-mutated, *NF1*-mutated and triple wild-type melanomas. This genetic classification may be useful for assessment of therapeutic options for patients with metastatic conjunctival melanoma

## Introduction

Conjunctival melanoma accounts for 5–10% of all ocular melanomas with a 10-year local recurrence rate of 38–69% and disease-related mortality of 13–38%.^1–6^ A better understanding of the genetics of conjunctival melanoma may help identify improved therapeutic options for patients with advanced disease.

In recent years, major melanoma subtypes have been genetically characterised. Cutaneous melanomas frequently harbour activating mutations in *BRAF* (~50%)^7^ or *NRAS* (~20%), as well as mutations in *NF1*.^8–11^ *BRAF*, *NRAS* and *NF1* mutations lead to activation of the mitogen-activated protein (MAP) kinase pathway.^9, 12, 13^ Based on these findings, a genetic classification of cutaneous melanomas has been proposed distinguishing four genetic groups: *BRAF*-mutated, *RAS*-mutated, *NF1*-mutated or triple wild type.^11^

Uveal melanomas exhibit a different mutation profile, and harbour mutations in *GNAQ*,^14^ *GNA11*,^15^ *CYSLTR2*,^16^ *PLCB4*,^17^ *EIF1AX*,^18^ *SF3B1*^19^ and *BAP1*,^20^ which are rarely found in other melanomas.^15, 21–23^

Conjunctival melanomas have not been characterised genetically as well as other melanoma subtypes. *BRAF*^*V600E*^ and *NRAS* mutations are present in 14–50%^24–27^ and 18%,^28^ respectively, of conjunctival melanomas. *TERT* promoter mutations were identified in 32–41% of conjunctival melanomas.^29, 30^ One study reported a *KIT* mutation in 1/14 (7%) tumours.^31^ Copy number analysis identified alterations reminiscent of cutaneous and mucosal melanomas, including *CDKN2A* and *PTEN* losses.^28^ These data suggest that conjunctival melanomas are genetically similar to cutaneous melanomas, but aside from *BRAF*, *NRAS* and *TERT* promoter mutations, recurrent mutations in other genes have not been identified.

There are two main therapeutic avenues for metastatic melanoma. Firstly, targeted small inhibitors dampening pathologically activated cell-intrinsic signalling mechanisms, with the most effective to date being a combination of BRAF and MEK inhibitors in *BRAF*-mutated melanoma.^32^ Secondly, immunotherapies applying anti-CTLA-4 and anti-PD-1 antibodies have shown impressive response rates in cutaneous and mucosal melanoma.^33–35^ Both approaches may be clinically useful in advanced conjunctival melanoma.^36^

Our study aimed to further elucidate genetic events in conjunctival melanoma by analysing a large tumour cohort with a targeted next-generation sequencing assay covering genes that are recurrently mutated in cutaneous and uveal melanoma.

## Materials and methods

### Sample selection and histopathology

Sixty-seven conjunctival melanoma samples were obtained from the tissue archives of the Departments of Ophthalmology, Dermatology and Pathology of the University Hospital Essen, and the Department of Ophthalmology, University Hospital Tübingen, Germany. The study was approved by the local ethics committee of the University of Duisburg-Essen.

### DNA isolation

Formalin-fixed, paraffin-embedded tumour tissues were sectioned, deparaffinised and manually microdissected as previously described.^37^ Genomic DNA was isolated using the QIAamp DNA Mini Kit (Qiagen, Hilden, Germany).

### Targeted sequencing

A custom amplicon-based sequencing panel covering 29 genes known to be mutated in melanoma was used (genes listed in Supplemental Table 1), as previously described.^37^ Mean coverage of 2094 reads, with a minimum coverage of 30 reads in >80% of the target loci, was achieved. Four samples were excluded from analysis due to low coverage.

### Sequence analysis

CLC Cancer Research Workbench from QIAGEN® was used for sequence analysis, as previously reported.^37^ Mutations were considered if coverage of the mutation site was ≥30 reads, ≥10 reads reported the mutated variant and the frequency of mutated reads was ≥10%.

### Associations of mutation status with clinical and pathological parameters

Associations of mutation status with available clinico-pathological parameters (listed in Table 1) were explored. Analyses were performed with IBM SPSS Statistics software (version 20.0; International Business Machines Corp., Armonk NY, USA). A *p*-value of <0.05 was considered statistically significant.

**Table 1:** Correlation between mutation status and clinical features in conjunctival melanomas

		Total		*BRAF* ^*WT*^		*BRAF* ^*V600E*^		*P*-value (*p* < 0.05)	*RAS* ^*WT*^		*RAS* ^*MUT*^		*P*-value (*p* < 0.05)	*NF1* ^*WT*^		*NF1* ^*MUT*^		*P-*value (*p* < 0.05)
		%	(*n*)	%	(*n*)	%	(*n*)		%	(*n*)	%	(*n*)		%	(*n*)	%	(*n*)	
Total			63	74.6	47	25.4	16		81.0	51	19.0	12		66.7	42	33.3	21	
Sex	Female	50.8	32	38.1	24	12.7	8	0.94	44.4	28	6.3	4	0.18	36.5	23	14.3	9	0.37
	Male	49.2	31	36.5	23	12.7	8		36.5	23	12.7	8		30.2	19	19	12	
Eye	Right	55.6	35	39.7	25	15.9	10	0.63	42.9	27	12.7	8	0.59	34.9	22	20.6	13	0.52
	Left	41.3	26	31.7	20	9.5	6		34.9	22	6.3	4		28.6	18	12.7	8	
	N/A	3.2	2	3.2	2	0	0		3.2	2	0	0		3.2	2	0	0	
TNM	1	55.6	35	41.3	26	14.3	9	0.77	44.4	28	11.1	7	0.44	38.1	24	17.5	11	0.56
	2	23.8	15	17.5	11	6.3	43		22.2	14	1.6	1		14.3	9	9.5	6	
	3	15.9	10	11.1	7	4.8	0		11.1	7	4.8	3		9.5	6	6.3	4	
	N/A	4.8	3	4.8	3	0			3.2	2	1.6	1		4.8	3	0	0	
Tumour origin	PAM	52.4	33	41.3	26	11.1	7	0.1	44.4	28	7.9	5	0.35	37.5	24	14.3	9	0.43
	Naevus	17.5	11	7.9	5	9.5	6		15.9	10	1.6	1		11.1	7	6.3	4	
	De novo	22.2	14	19	12	3.2	2		15.9	10	4.8	4		11.1	7	11.1	7	
	N/A	7.9	5	6.3	4	1.6	1		4.8	3	3.2	2		6.3	4	1.6	1	
Relapses	No	46	29	36.5	23	9.5	6	0.26	33.3	21	12.7	8	0.22	27	17	19	12	0.46
	Yes	47.6	30	31.7	20	15.9	10		42.9	27	4.8	3		34.9	22	12.7	8	
	N/A	6.3	4	6.3	4	0	0		4.8	3	1.6	1		4.8	3	1.6	1	
Metastasis	No	68.3	43	49.2	31	19.0	12	0.32	54.0	34	14.3	9	0.34	42.9	27	25.4	16	0.55
	Yes	22.2	14	15.9	10	6.3	4		20.6	13	1.6	1		17.5	11	4.8	3	
	N/A	9.5	6	9.5	6	0	0		6.3	4	3.2	2		6.3	4	3.2	2	
Exenteration	No	76.2	48	57.1	36	19.0	12	0.36	60.3	38	15.9	10	0.64	47.6	30	28.6	18	0.44
	Yes	17.5	11	11.1	7	6.3	4		15.9	10	1.6	1		14.3	9	3.2	2	
	N/A	6.3	4	6.3	4	0	0		4.8	3	1.6	1		4.8	3	1.6	1	
Age at diagnose	Median 67.4 years , Range 40.1–88.8 years																	

Clinical and pathological stage is according to TNM 7th edition AJCC 2010 for conjunctival melanoma

*N/A* not assessable, *PAM* primary acquired melanosis

## Results

### Tumours and patients

Conjunctival melanomas occurred equally in male and female with a median age of 67 years (range 40–89 years). Of the samples for which information was available, 52% (33/63) originated from primary acquired melanosis (PAM), 18% (11/63) from naevi and 22% (14/63) arose de novo. Clinical stage at initial presentation was stage 1, stage 2 and stage 3 in 56% (35/63), 24% (15/63) and 16% (10/63) of patients, respectively (American Joint Committee on Cancer staging system for conjunctival melanoma, 7th edition, 2010). Adjuvant treatment was received by 87% (55/63) of patients (21 ruthenium, 17 proton, 6 percutaneous radiotherapy, 7 cryotherapy and 3 mitomycin C). Tumours recurred in 47% (30/63) and metastasised in 22% (14/63) of cases. Additional information is listed in Table 1.

### Identified mutations

Activating *BRAF*^*V600E*^ (c.1799A>T) mutations were detected in 16/63 (25%) tumours. Additionally, 4 *BRAF* mutations with unknown functional consequences were identified (Supplemental Table 2).

Activating *RAS* mutations (11 *NRAS* and 1 *KRAS* mutation) were identified in 12/63 (19%) tumours (Table 2). We also detected 4 *NRAS*, 3 *KRAS* and 5 *HRAS* mutations with unknown functional consequences (Supplemental Table 2).

**Table 2:** MAP kinase pathway activating mutations in conjunctival melanoma

Gene		Mutation type	Tumours harbouring mutation	
			*N*	%
*BRAF*		All mutations	16	25
		V600E	16	25
*RAS*		All mutations	12	19
	NRAS	Q61R	5	8
		Q61K	2	3
		Q61H	1	2
		Q61L	1	2
		G13D	1	2
		G12N, G12C	1	2
	KRAS	G12A	1	2
*NF1*		All mutations	21	33
		T60del	1	2
		R262C	1	2
		C42Y, G2397R, S2587L	1	2
		S2751N, L552P, G2392E	1	2
		D176E	2	3
		L847P, P866S, V1762I	1	2
		C1899Y	1	2
		M1180I, S52F; T60I	1	2
		A2715V; A2208T	1	2
		G2397R, R2517fs	1	2
		I1824fs	1	2
		L1892^a^	1	2
		N1451L	1	2
		Q1815^a^	1	2
		Q756fs	1	2
		R1362^a^	1	2
		R440^a^, Q2239^a^; S1497F; V1393A	1	2
		S168L	1	2
		S1786^a^, L1102^a^; Q1815fs	1	2
		Y1678fs	1	2
Wild type			14	22
Total			63	

*MAP* mitogen-activated protein, *fs* frameshift mutations

^a^Nonsense mutations

**Fig. 1:**
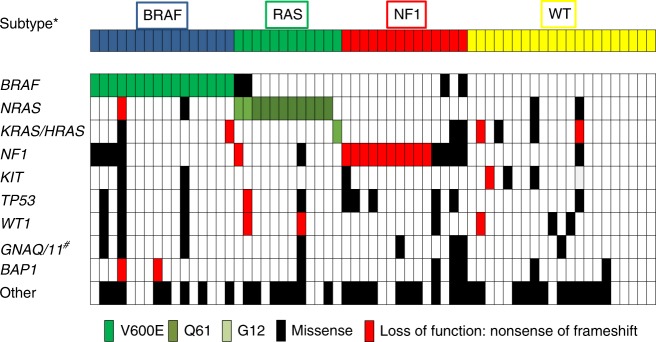
Mutations in conjunctival melanoma. Distribution of mutations identified by amplicon panel next-generation sequencing. Green: mutations known or assumed to be activating; Red: nonsense or frameshift loss-of-function mutations; Black: missense mutation with unknown functional consequences. Mutations listed as “Other” include mutations detected in *CDK4*, *FLT4*, *PIK3CA*, *PIK3R1*, *FBXW7*, *MITF*, *MAP2K1*, *MAP2K2*, *ARID1A*, *ARID2*, *SF3B1*, *CTNNB1*, *PTEN*, *CDKN2A*, *SMARCA4A*, *EZH2*, *IDH1* and the protein-coding area of *TERT* (the promoter region of *TERT* was not covered by the amplicon-based sequencing panel used in this study). *Subtype according to TCGA genomic classification of cutaneous melanoma. ^#^None of the *GNAQ* or *GNA11* mutations identified were the known activating Q209 or R183 mutations recurrently identified in uveal melanomas (details in Supplemental Table 2)

*NF1* mutations were identified in 21/63 (33%) tumours. Clearly inactivating *NF1* mutations were observed in 10 tumours. *NF1* mutations co-occurred with *BRAF* and *RAS* gene mutations in some tumours, but also frequently occurred alone. All identified mutations are shown in Fig. 1 and listed in Table 2, and shown in Supplemental Figs. 1 and 2.

Additionally, mutations in various genes frequently mutated in cutaneous melanoma were detected. The majority of these mutations were of unknown functional consequences (Supplemental Table 2). While a few *GNAQ* and *GNA11* mutations were identified (Fig. 1, Supplemental Table 2), they presumably represent functionally non-relevant bystander mutations, as none of the identified mutations were the activating R183 or Q209 mutations known to occur in uveal melanomas.^14, 15, 21^

### Statistical analysis

There were no statistically significant associations between clinico-pathological parameters with *BRAF*, *RAS* and *NF1* mutation status (Table 1).

## Discussion

To our knowledge, the present study represents the most detailed analysis of gene mutations in conjunctival melanoma to date.

Activating *BRAF* mutations were detected in 25% of samples, lying within the range of previous studies reporting 14–50%.^24–27, 38^ This variation may be due to sample bias or technical differences. In view of the recent development of effective BRAF and MEK inhibitors, the presence of *BRAF* V600 mutations in conjunctival melanomas is of considerable therapeutic relevance.^39^

In addition to known activating *NRAS* mutations in 18% (11/63) of tumours, we identified an activating *KRAS* G12A mutation. Being the first report on these mutations in conjunctival melanoma, this finding is reminiscent of cutaneous melanoma, in which *KRAS* mutations are rare but occur in a mutually exclusive fashion with *NRAS* mutations.^11^ In the proposed TCGA (The Cancer Genome Atlas) genomic classification of cutaneous melanoma, mutations in all three *RAS* genes are grouped together as *RAS*-mutated melanomas.

Our study is the first to identify *NF1* as a frequently mutated oncogene (33%) in conjunctival melanoma. *NF1* has recently been recognised as the third most commonly mutated gene (after *BRAF* and *RAS*) in cutaneous melanoma, activating the MAP kinase pathway.^11^ In our conjunctival melanoma cohort, *NF1* mutations were also present in samples harbouring activating *RAS* or *BRAF* mutations (Fig. 1). This is similar to the situation in cutaneous melanoma where the co-occurrence of *NF1* with *BRAF*, *RAS* and other mutations is well recognised.^11, 12, 40^

*NF1* mutations are particularly frequent in melanoma subtypes rarely harbouring *BRAF* and *NRAS* mutations,^8, 12, 41^ including melanomas associated with high sun exposure.^8, 12^ Ultraviolet exposure is a known pathogenic factor in conjunctival melanoma and could explain the high number of *NF1* mutations detected. *NF1* mutations have been associated with high tumour mutational load and affected patients have been reported to benefit from anti-PD-1 therapy in cutaneous melanoma.^42^ This suggests that *NF1* mutation status has potential as a biomarker for immunotherapy in conjunctival melanoma.

In summary, our study identifies a range of mutations in conjunctival melanoma. The distribution of activating mutations, with *RAS* gene mutations occurring not only in *NRAS* but also *KRAS*, and *NF1* mutations being frequent in tumours lacking *BRAF* or *RAS* mutations, suggests that the proposed genetic classification of cutaneous melanomas into *BRAF*-mutated, *RAS*-mutated, *NF1*-mutated or triple-wild-type tumours is also applicable to conjunctival melanoma.

## Electronic supplementary material

Supplementary Figure 1

Supplementary Figure 2

Supplementary Table 1

Supplementary Table 2

Supplementary Information
