# Supplementary figures and images for: NF1 mutations in conjunctival melanoma
Source: Br J Cancer. 2018 Mar 21;118(9):1243–7. doi: 10.1038/s41416-018-0046-5 (PMC5943412; doi:10.1038/s41416-018-0046-5)

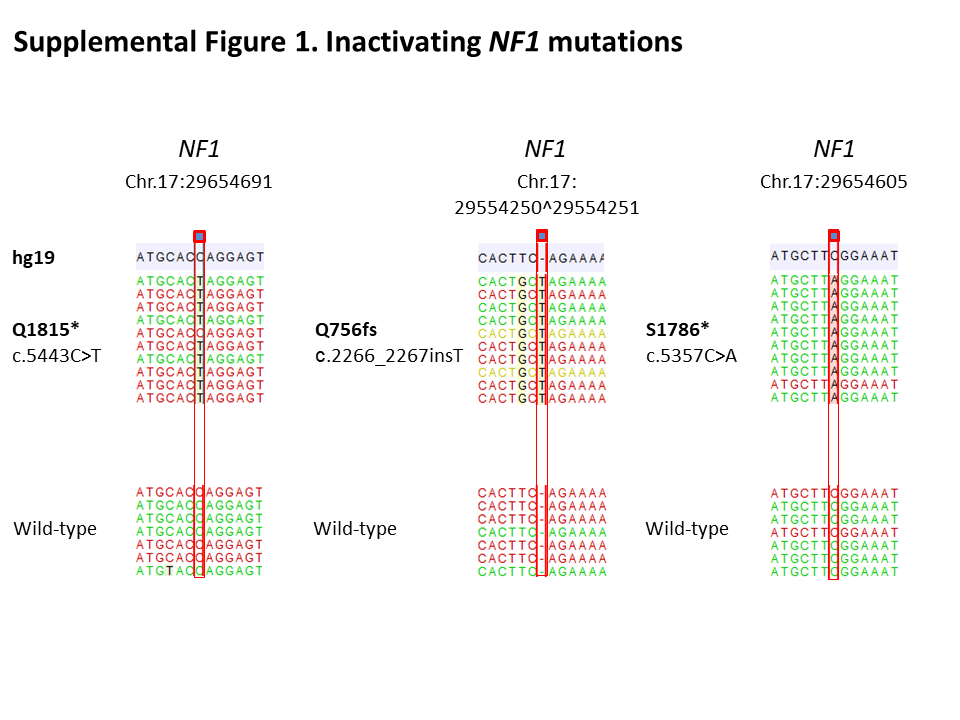

Supplement: Supplementary file 1 — Supplementary Figure 1 [file 41416_2018_46_MOESM1_ESM.tif]

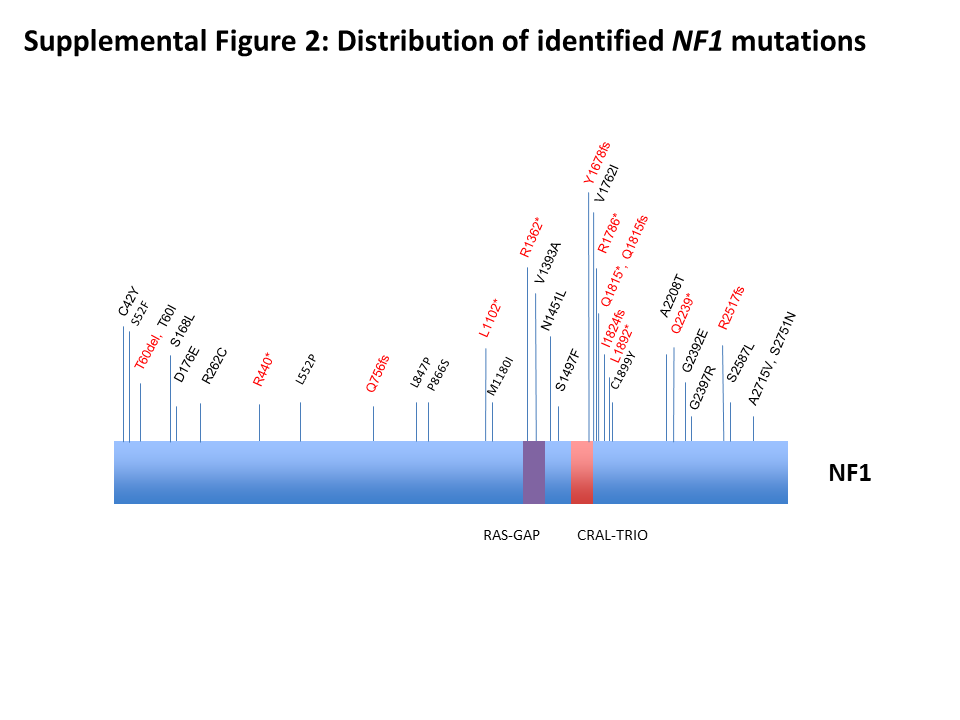

Supplement: Supplementary file 2 — Supplementary Figure 2 [file 41416_2018_46_MOESM2_ESM.tif]
