# Supplementary material for: NF1 mutations in conjunctival melanoma
Source: Br J Cancer. 2018 Mar 21;118(9):1243–7. doi: 10.1038/s41416-018-0046-5 (PMC5943412; doi:10.1038/s41416-018-0046-5)
Supplement: Supplementary file 3 — Supplementary Table 1 [file 41416_2018_46_MOESM3_ESM.docx]

| **Nr.** | **Gene** | **Primary melanoma type** | **Customary mutation type** | **Target bases** | **Bases covered** | **Primer pairs** |
| --- | --- | --- | --- | --- | --- | --- |
| 1 | BRAF | cutaneous | activating | 2860 | 2456 | 40 |
| 2 | NRAS | cutaneous | activating | 650 | 650 | 10 |
| 3 | KIT | cutaneous | activating | 3354 | 3264 | 51 |
| 4 | HRAS | cutaneous | activating | 780 | 667 | 11 |
| 5 | KRAS | cutaneous | activating | 787 | 787 | 13 |
| 6 | CDKN2A | cutaneous | tumour suppressor | 1184 | 713 | 14 |
| 7 | PTEN | cutaneous | tumour suppressor | 1392 | 1248 | 22 |
| 8 | CDK4 | cutaneous |  | 1052 | 1052 | 19 |
| 9 | TP53 | cutaneous | tumour suppressor | 1503 | 1396 | 26 |
| 10 | RAC1 | cutaneous |  | 776 | 721 | 14 |
| 11 | NF1 | cutaneous | tumour suppressor | 9900 | 9167 | 143 |
| 12 | PIK3CA | cutaneous |  | 3607 | 3313 | 50 |
| 13 | MAP2K2 | cutaneous |  | 1423 | 1240 | 24 |
| 14 | PIK3R1 | cutaneous |  | 2637 | 2627 | 42 |
| 15 | MITF | cutaneous |  | 2066 | 2066 | 35 |
| 16 | TERT* | cutaneous |  | 3719 | 2371 | 39 |
| 17 | ARID2 | cutaneous | tumour suppressor | 5928 | 5830 | 82 |
| 18 | ARID1A | cutaneous | tumour suppressor | 7258 | 6132 | 81 |
| 19 | SMARCA4 | cutaneous | tumour suppressor | 5761 | 5040 | 88 |
| 20 | MAP2K1 | cutaneous |  | 1436 | 1436 | 26 |
| 21 | CTNNB1 | cutaneous |  | 2626 | 2626 | 40 |
| 22 | EZH2 | cutaneous |  | 2680 | 2680 | 46 |
| 23 | IDH1 | cutaneous |  | 1405 | 1394 | 22 |
| 24 | FBXW7 | cutaneous |  | 2898 | 2808 | 43 |
| 25 | WT1 | cutaneous |  | 1784 | 1282 | 24 |
| 26 | GNAQ | uveal | activating | 1220 | 1064 | 17 |
| 27 | GNA11 | uveal | activating | 1220 | 944 | 14 |
| 28 | BAP1 | uveal | tumour suppressor | 2599 | 2380 | 39 |
| 29 | SF3B1 | uveal |  | 4455 | 4412 | 72 |

* the protein-coding area (but not the promoter region) of the *TERT* gene was covered by the panel.
