# Supplementary material for: NF1 mutations in conjunctival melanoma
Source: Br J Cancer. 2018 Mar 21;118(9):1243–7. doi: 10.1038/s41416-018-0046-5 (PMC5943412; doi:10.1038/s41416-018-0046-5)
Supplement: Supplementary file 5 — Supplementary Information [file 41416_2018_46_MOESM5_ESM.docx]

**SUPPLEMENTAL FIGURE LEGENDS**

**Supplemental Figure 1: Examples of inactivating *NF1* mutations**

Shown are the results of 3 samples in which inactivating mutations were detected by amplicon-based next-generation sequencing. Demonstrated on top are the sequenced results where a nucleotide alteration was determined. A wild-type result from another sample is shown underneath for comparison.

**Supplemental Figure 2. Distribution of identified *NF1* mutations**

Demonstrated a scheme of the NF1 protein with the mutations identified at different locations in the protein. Functionally inactivating mutations leading to a premature stop-codon (non-sense) or frameshift are demonstrated in red.

**SUPPLEMENTAL TABLES**

**Supplemental Table 1: Genes covered in the applied sequencing panel**

**Supplemental Table 2: List of all mutations for each tumour sample**
